# Association between physical activity and psychological status among Saudi female students

**DOI:** 10.1186/s12888-014-0238-3

**Published:** 2014-08-21

**Authors:** Einas Al-Eisa, Syamala Buragadda, Ganeswara Rao Melam

**Affiliations:** Female Centre for Science and Medical Studies, King Saud University, Riyadh, Saudi Arabia; Rehabilitation Health Sciences Department, College of Applied Medical Sciences, King Saud University, Riyadh, Saudi Arabia

**Keywords:** Physical performance, Pedometer, Depression, Insomnia, Attention

## Abstract

**Background:**

Physical inactivity is common among Saudi females. Many variables are associated with different levels of mental health, including physical activity. This study was designed to determine the correlation between 3 weeks of improved physical activity and psychological factors such as insomnia, depression and attention span.

**Methods:**

Seventy-six female students, of mean age 20.9 ± 1.4 years, were analyzed. Insomnia, depression and attention were subjectively assessed using the Insomnia Severity Index (ISI), the Beck Depression Inventory (BDI), and the Attention Span Test (AST), respectively. Each subject was given a pedometers and advised to walk at least 6000 steps per day for 3 weeks. Psychological status was assessed before and after the 3 weeks and compared using paired sample t-tests. Pearson correlation was used to determine the association between physical health and psychological factors.

**Results:**

Improvements in scores on the ISI (from 7.22 ± 3.06 to 4.09 ± 2.80), BDI (from 8.88 ± 3.13 to 3.98 ± 2.74) and AST (from 63.86 ± 3.06 to 77.27 ± 11.33) were observed after 3 weeks. Physical activity was negatively correlated with ISI (r = −0.74) and BDI (r = −0. 78) and positively correlated with AST (r = 0.69).

**Conclusion:**

Improved physical activity can be useful in managing insomnia, depression and attention. In female Saudi students, higher levels of physical activity were associated with improved mental health.

## Background

Many populations based studies have shown that physical activity has many beneficial effects on mental health [1]. Physical activity is measured in different domains, including type, frequency, duration, intensity and relative importance in achieving specific outcomes [2]. According to the World Health Organization (WHO), physical inactivity leads to disability and is one of the leading causes of chronic conditions such as hypertension, diabetes, and obesity [3]. People with low physical activity are at higher risk of exhibiting symptoms of depression and anxiety. Mood disorders are also associated with physical inactivity and increased physical activity helps to improve mood disturbances. Studies have shown an inverse relationship between physical activity and depression [4].

Physical inactivity is common among Saudis of all age groups, with prevalences ranging from 43 to 99%. Saudi males frequently participate in vigorous physical activity, whereas Saudi females do not [5]. Physical activity in adolescents improves brain function and cognitive performance and may help reduce stress and mood alterations, reductions that can improve academic performance in school. Psychological and emotional disorders play a major role in the performance of college students [6-8]. Self-determined walking for 30 minutes per day, even in short bouts of 10 minutes each, has been regarded as moderate to vigorous physical activity (MVPA) [9]. Pedometers are the most reliable and valid tools for measuring and promoting physical activity. These instruments can measure the number of steps taken and record the amount of time a person is active and the number of calories expended. Steps per minute (SPM) can be calculated by dividing the total steps taken by the total activity time in minutes [10,11]. An SPM of 120–140 has been recommended for adolescents aged 10–12 years, whereas 30 minutes of MVPA or 10,000 steps per day on all weekdays is recommended for adults [12,13]. For Chinese adults, the step rate cut point at 6 METs (metabolic equivalents) is 130 steps per minute, and the recommended number of steps per day is 3150 [14]. Incorporating a device such as a pedometer can increase an individual’s awareness and motivation to change his/her sedentary lifestyle [10,15].

Physical inactivity has an adverse impact on health of all individuals including college students [16-18]. Lifestyle behaviors play an important role in healthy living. Poor mental wellbeing is often expressed as low self-esteem, depressed mood, lack of self-confidence, insomnia, and social isolation. According to the WHO, the second most frequent cause of disability by 2020 will be mental illness, specifically depression [19]. Insomnia is highly prevalent among college students, leading to stress, depression and reduced academic performance. Sleep deprivation and psychological stress are more frequently observed in medical students in various countries. Moreover, physical activity was shown to be negatively correlated with insomnia. The Insomnia Severity Index (ISI) is a valid and reliable tool for measuring insomnia [20-22]. Depression and depressive disorders are considered public health burdens, with 10–15% of youths experiencing a mood disturbance at any one time. In adults, the risk of depression can be reduced by increasing physical activity [23]. The type, intensity and frequency of physical activity for effective stress control remains unclear. Leisure time physical activity leads to good mental health in women [24], with physical activity having beneficial effects on brain structure and function [25]. Students involved in physical activity develop greater self-esteem, increased attention and better classroom behavior [26]. Much work is required to determine the correlation between physical activity and mental health among Saudi female college students. This study was therefore designed to determine the correlation between a 3-week physical activity motivation program and mental health variables, including attention, depression and insomnia, in female Saudi students.

## Methods

### Sample

Female students at the Applied Medical Sciences College of King Saud University were invited to attend a 1-hour lecture on the adverse effects of physical inactivity and its prevalence in Saudi Arabia. Of those who attended, 105 volunteered to participate, but only 76 completed the study. Students ranged in age from 19 to 25 years. Subjects with a history of fracture or surgery of the back, pelvis, or lower limb, any contraindication to increased walking, pregnancy, or any cognitive or communication impairment were excluded. All participants provided written informed consent, and the study protocol was approved by the Ethics Committee of King Saud University.

The next presentation was given only to participants; this included the outline of the study, including its purpose and methods, how to use the pedometer, and the effects of exercise. A leaflet containing several simple suggestions for increasing physical activity was given to each participant. Examples included walking around while talking on a cell, using stairs instead of an elevator, and going window shopping at least once a week. Demographic data, such as age, height, weight, and body mass index (BMI), were recorded. Self-administered questionnaires were used to measure attention (AST), insomnia (ISI), and depression (BDI). Each subject was given a pedometer and instructed to walk at least 6000 steps per day for 3 weeks. Stride length was determined for each subject by marking a taped 7-meter distance on the floor and instructing each subject to take 10 steps forward (five steps with each foot) at their normal stride; stride length was calculated by dividing the distance covered by 10. Subjects were instructed to wear the pedometer (Omron model HJ – 152) at the level of the anterior superior iliac spine (ASIS). Communication was maintained through email and telephone to solve any problems encountered. Subjects were asked to maintain a daily physical activity log for 3 weeks. At the end of each week, each participant was sent an e-mail and telephone reminder to maintain their daily physical log and to improve their physical activity.

The AST consists of 10 questions and takes ≤5 minutes to complete. Score ranges from 0 to 100, with higher scores indicating a better attention span and better ability to focus on a task with no distractions. The Beck Depression Inventory (BDI), a self-reported questionnaire used to measure depression, has been shown to be useful in both research and clinical practice. The most recent form of the BDI, the BDI-II (second version), was found to better differentiate depressed from non-depressed subjects than the original BDI [27,28]. The BDI consists of 21 items, with scores ranging from 0 to 63. Scores of 1–10 and 11–16 were considered normal and indicative of mild mood disturbances, respectively; scores of 17–20, 21–31, 31–40, and >40 were considered indicative of borderline, moderate, severe, and extreme depression, respectively. The insomnia severity index (ISI) consists of seven questions, with scores ranging from 0 to 28; scores of 0–7 indicated an absence of clinically significant insomnia; 8–14 indicated sub-threshold insomnia; and scores of 15–21 and 22–28 indicating moderate and severe clinical insomnia, respectively.

### Statistical analysis

Data were analyzed using SPSS-16. Differences in physical activity from before to after the 3-week period were analyzed using one way ANOVA. Scores on each of the three questionnaires before and after the 3-week period were compared using paired sample t-tests. The relationships between physical activity and insomnia, depression and attention were assessed by Pearson correlation analysis. A p value ≤ 0.05 was considered statistically significant.

## Results

Of the 105 students initially recruited, 29 dropped out before or during the study for various reasons. Finally, 76 subjects completed the study successfully. Their mean age was 20.9 ± 1.4 years; their mean weight was 59.5 ± 12.9 kg, their mean height was 157.9 ± 6.2 cm, and their mean was BMI 23.8 ± 4.8 kg/m^2^.

**Figure 1:**
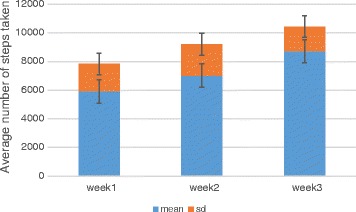
**Physical activity (average number of steps taken) from week 1 to 3.**

The mean numbers of steps taken by the study participants after 1, 2 and 3 weeks of physical activity were 5917, 7026, and 8715, respectively (Figure 1). Before the 3-week physical activity period, 52% of study subjects had mild mood disturbances (scores 11–16 on the BDI), with the average pre-treatment score on the BDI of 8.88 ± 3.13. Their average score on the ISI was 7.22 ± 3.06 indicating sub threshold insomnia; and their average score on the AST was 63.85 ± 11.18, indicating a lack of focus and attention on a task. Following the 3-week physical activity period, the subjects had mean scores on the BDI, ISI, and AST of 3.98 ± 2.74 (t = 10.22, P ≤ 0.05), 4.09 ± 2.80 (t = 6.57, P ≤ 0.05), and 77.27 ± 11.33 (t = −7.34, P ≤ 0.05), respectively, with all showing significant Improvements after 3 weeks.

**Figure 2:**
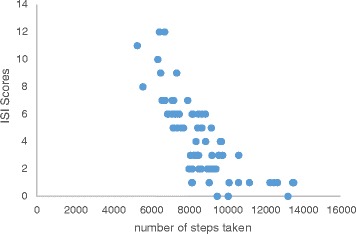
**Correlation analysis between physical activity and insomnia.** *negative correlation was observed between ISI scores and number of steps taken.

**Figure 3:**
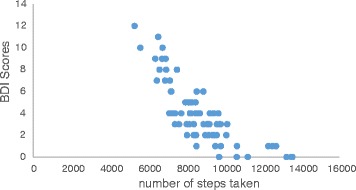
**Correlation analysis between physical activity and depression.** *negative correlation was observed between BDI scores and number of steps taken.

**Figure 4:**
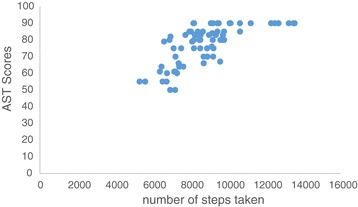
**Correlation analysis between physical activity and attention.** *Positive correlation was observed between AST scores and number of steps taken.

Pearson correlation analysis showed that physical activity was negatively correlated with ISI (r = −0.74; p ≤ 0.01; Figure 2) and BDI (r = −0. 78; p ≤ 0.01; Figure 3) and positively correlated with AST (r = 0.69; p ≤ 0.01; Figure 4). Further analysis showed a positive correlation between ISI and BDI (r = 0. 77; p ≤ 0.01), a negative correlation between ISI and AST (r = −0.77; p ≤ 0.01), and a negative correlation between BDI and AST (r = −0.68; p ≤ 0.01).

## Discussion

This study was designed to determine the correlation between physical activity and psychological factors, such as attention, depression and insomnia, in young Saudi women (mean age, 20.9 ± 1.4 years) who were not overweight (mean weight, 59.5 ± 12.9 kg; mean BMI 23.8 ± 4.8 kg/m^2^). Adherence and dropout are the two most important components related to physical inactivity, with an average dropout rate of 50% [29]. Non-adherence has been associated with barriers to exercise, perceived lack of time, influence by family and peers, and lack of will power [30]. In contrast, the dropout rate in our study was only 30%, with dropout likely due to a lack of time, stress from examinations and lack of motivation. Although leisure time physical activity is associated with a lower incidence of mental disorders [24,25,31], the dose–response relationship between exercise and mental well-being is still unclear [32]. Moderate or vigorous physical activity is recommended to improve health and reduce the risk of chronic illness when performing the routine activities of daily living does not meet the recommended dosage of physical activity [33]. Minimum activity guidelines vary for individuals in different countries. It has been estimated that half of the US adult population does not meet the recommended guideline [34,35]. Moreover, Australian adults walking <7500 steps were considered “inactive” [36].

To our knowledge, there have been no such recommendations for young Saudis. We found, however, that physical activity was associated with psychological factors, such as insomnia, depression and attention span. Exercise of any type was shown to have positive effects on depression [37]. Physical activities were found to be negatively associated with mental disorders, especially anxiety and mood disturbance [38]. We observed that physical activity was negatively correlated with insomnia (r = −0.74) and depression (r = −0.78). Insomnia can lead to daytime sleepiness and inattention, resulting in frequent attention shifting and distraction [39]. Our finding that exercise for 3 weeks improved ISI and BDI scores was consistent with results showing that increased physical activity reduced depression (t = 10.22, p ≤ 0.05) and insomnia (t = 6.57, p ≤ 0.05) symptoms [40]. We also observed a positive correlation between physical activity and attention span (r = 0.69). Depression and insomnia influence attention, with distraction and an inability to focus on a task reducing subject productivity [41]. This is also consistent with our results, which showed that depression (r = −0.68) and insomnia (r = −0.77) were negatively correlated with attention. The findings of this study show that Saudi female students who exercise regularly are less likely to experience mental disturbances. The rate of response was 70%. The sample size was small and the duration of the physical exercise program was only 3 weeks. The motivational program was not structured. Future research should focus on a larger sample, with longitudinal studies recommended.

## Conclusion

To our knowledge this is the first study analyzing the relationship between physical activity and psychological factors in Saudi female students. Improved physical activity can improve symptoms of insomnia and depression and improve the ability to focus on a particular task without distraction.

## References

[1] Mata J, Thompson RJ, Jaeggi SM, Buschkuehl M, Jonides J, Gotlib IH (2012). Walk on the bright side: Physical activity and its affect in major depressive disorder. J Abnorm Psychol.

[2] Melinda A, Katrien W, De Bourdeaudhuij I, Philippaerts R, Matton L, Duviqneaud N, Martine T, William D, Johan L, Greet C (2009). Specific associations between types of physical activity and components of mental health. J Sci Med Sport.

[3] ᅟ (2001). Sedentary lifestyle: a global public health problem. ᅟ.

[4] De Mello MT, Lemos Vde A, Antunes HK, Bittencourt L, Santos-Silva R, Tufik S (2013). Relationship between physical activity and depression and anxiety symptoms: A population study. J Affect Dis.

[5] Khalaf A, Ekblom Ö, Kowalski J, Berggren V, Westergren A, Al-Hazzaa H (2013). Female university Students’ physical activity levels and associated factors—a cross-sectional study in southwestern Saudi Arabia. Int J Environ Res Public Health.

[6] Kantomaa MT, Tammelin TH, Demakakos P, Ebeling HE, Taanila AM (2010). Physical activity, emotional and behavioral problems, maternal education and self-reported educational performance of adolescents. Health Educ Res.

[7] Hillman CH, Erickson KI, Kramer AF (2008). Be smart, exercise your heart: exercise effects on brain and cognition. Nat Rev Neurosci.

[8] Kessler RC, Foster CL, Saunders WB, Stang PE (1995). Social consequences of psychiatric disorders, I: educational attainment. Am J Psychiatry.

[9] Murtagh EM, Boreham CA, Murphy MH (2002). Speed and exercise intensity of recreational walkers. Prev Med.

[10] Susan VG, Robert PP, William JV (2009). Step it up: Activity intensity using pedometers. JOPERD.

[11] Tudor-Locke CE (2002). Taking steps toward increased physical activity: using pedometers to measure and motivate. Res Dig.

[12] Behrens T, Hawkins S, Dinger M (2005). Relationship between objectively measured steps and time spent in physical activity among free-living college students. Meas Phys Educ Exerc Sci.

[13] Choi BC, Pak AW, Choi JC, Choi EC (2007). Daily step goal of 10,000 steps: A Literature review. Clin Invest Med.

[14] Wang H, Zhang YF, Xu LL, Jiang CM (2013). Step rate-determined walking intensity and walking recommendation in Chinese young adults: a cross-sectional study. BMJ.

[15] Tudor-Locke C, Cora LC, John PT, John CS (2012). A step-defined sedentary lifestyle index: <5000 steps/day. Appl Physiol Nutr Metab.

[16] Craig CL, Shields M, Leblanc AG, Tremblay MS (2012). Trends in aerobic fitness among Canadians, 1981 to 2007–2009. Appl Physiol Nutr Metab.

[17] Hackmann Debra J, Mintah JK (2010). Pedometers: a strategy to promote increased physical activity among college students. J Instruct Pedagogies.

[18] Behrens T, Dinger M (2003). A preliminary investigation of college students’ physical activity patterns. Am J Health Stud.

[19] Melinda A, Katrien W, de Bourdeaudhuij I, Philippaerts R, Matton L, Duvigneaud N, Martine T, Johan L, Greet C (2012). Sport participation and stress among women and men. Psychol Sport Exerc.

[20] Angelone AM, Mattei A, Sbarbati M, Di Orio F (2011). Prevalence and correlates for self-reported sleep problems among nursing students. J Prev Med Hyg.

[21] Sing CY, Wong WS (2011). Prevalence of insomnia and its psychosocial correlates among college students in Hong Kong. J Am Coll Health.

[22] Yoshitaka Kaneita MD, Takashi Ohida MD, Yoneatsu Osaki MD (2006). Insomnia among Japanese adolescents: a nationwide representative survey. Sleep.

[23] McKercher C, Schmidt MD, Sanderson K, Dwyer T, Venn AJ (2005). Physical activity and depressed mood in primary and secondary school- children. Mental Health Phys Act.

[24] Ohta M, Mizoue T, Mishima N, Ikeda M (2007). Effect of physical activities in leisure time and commuting to work on mental health. J Occup Health.

[25] Berwid OG, Jeffrey MH (2013). Emerging support for role of exercise in Attention Deficit/Hyper activity Disorder intervention planning. Curr Psychiatry Rep.

[26] Shephard RJ (1997). Curricular physical activity and academic performance. Pediatr Exerc Sci.

[27] Richter P, Werner J, Heerlein A, Kraus A, Sauer H (1998). On the validity of the beck depression inventory. A review. Psychopathology.

[28] Arnarson TO, Olason DT, Smari J (2008). The Beck Depression Inventory Second Edition (BDI-II): psychometric properties in Icelandic student and patient populations. Nord J Psychiatry.

[29] Dishman RK, Buckworth J (1996). Increasing Physical activity a quantitative synthesis. Med Sci Sport Exerc.

[30] Rod KD, Sallis JF, Orenstein DR (1985). The determinants of physical activity and exercise. Public Health Rep.

[31] Rovio S, Kareholt I, Helkala E-L, Viitanen M, Winblad B, Tuomilehto J, Soininen H, Nissinen A, Kivipelto M (2005). Leisure-time physical activity at midlife and the risk of dementia and Alzheimer’s disease. Lancet Neurol.

[32] Panteleimon E, Erik L, Roxane RJ (2006). Can self reported preference for exercise intensity predict physiologically defined self selected exercise intensity?. Res Q Exerc Sport.

[33] Raphael B, Jeffry AJ, Ronald CP (2007). Physical activity level and health-related quality of life in the general adult population: A systematic review. Prev Med.

[34] Pate RR, Pratt M, Blair SN, William LH, Caroline AM, Claude B, David B, Walter E, Gregory W, Abby CK, Andrea K, Arthur SL, Marcus BH, Morris J, Ralph SP, Patrick K, Michael LP, Rippe JM, Sallis J, Wilmore JH (1995). Physical activity and public health: a recommendation from the centers for disease control and prevention and the American college of sports medicine. JAMA.

[35] Thompson DL, Rakow J, Perdu SM (2004). Relationship between accumulated walking and body composition in middle-aged women. Med Sci Sport Exerc.

[36] Tudor-Locke C, Giles Corti B, Knuiman M, McCormack G (2008). Tracking of pedometer-determined physical activity in adults who relocate: results from RESIDE. Int J Behav Nutr Phys Act.

[37] Lawlor DA, Hopker SW (2001). The effectiveness of exercise as an intervention in the management of depression: systematic controlled trials. BMJ.

[38] ten Have M, de Graaf R, Monshouwer K (2011). Physical exercise in adults and mental health status. Findings from the Netherlands Mental Health Survey and Incidence Study (NEMESIS). J Psychosom Res.

[39] Gau SS, Kessler RC, Tseng WL, Wu YY, Chiu YN, Yeh CB, Hwu HG (2007). Association between sleep problems and symptoms of attention-deficit/ hyperactivity disorder in young adults. Sleep.

[40] Karen P, Lucy Y, Tony K (2012). Physical activity and depression: A multiple median analysis. Mental Health Phys Act.

[41] Andrews PW, Aggen SH, Miller GF, Radi C, Dencoff JE, Neale MC (2007). The functional design of depression’s influence on attention: A preliminary test of alternative control-process mechanisms. Evol Psychol.

